# Evolution of vacuolar proton pyrophosphatase domains and volutin granules: clues into the early evolutionary origin of the acidocalcisome

**DOI:** 10.1186/1745-6150-6-50

**Published:** 2011-10-05

**Authors:** Manfredo J Seufferheld, Kyung Mo Kim, James Whitfield, Alejandro Valerio, Gustavo Caetano-Anollés

**Affiliations:** 1 Department of Crop Sciences, University of Illinois at Urbana-Champaign, Urbana IL USA; 2 Evolutionary Bioinformatics Laboratory, Department of Crop Sciences, University of Illinois at Urbana-Champaign, Urbana IL USA; 3 Korean Bioinformation Center (KOBIC), Korea Research Institute of Bioscience and Biotechnology (KRIBB), Daejeon 305-806, Korea; 4 Department of Entomology, University of Illinois at Urbana-Champaign, Urbana IL, USA; 5 Department of Evolution, Ecology and Organismal Biology, Museum of Biological Diversity, 1315 Kinnear Rd., Columbus, OH 43210, USA

## Abstract

**Background:**

Volutin granules appear to be universally distributed and are morphologically and chemically identical to acidocalcisomes, which are electron-dense granular organelles rich in calcium and phosphate, whose functions include storage of phosphorus and various metal ions, metabolism of polyphosphate, maintenance of intracellular pH, osmoregulation and calcium homeostasis. Prokaryotes are thought to differ from eukaryotes in that they lack membrane-bounded organelles. However, it has been demonstrated that as in acidocalcisomes, the calcium and polyphosphate-rich intracellular "volutin granules (polyphosphate bodies)" in two bacterial species, *Agrobacterium tumefaciens*, and *Rhodospirillum rubrum*, are membrane bound and that the vacuolar proton-translocating pyrophosphatases (V-H^+^PPases) are present in their surrounding membranes. Volutin granules and acidocalcisomes have been found in organisms as diverse as bacteria and humans.

**Results:**

Here, we show volutin granules also occur in Archaea and are, therefore, present in the three superkingdoms of life (Archaea, Bacteria and Eukarya). Molecular analyses of V-H^+^PPase pumps, which acidify the acidocalcisome lumen and are diagnostic proteins of the organelle, also reveal the presence of this enzyme in all three superkingdoms suggesting it is ancient and universal. Since V-H^+^PPase sequences contained limited phylogenetic signal to fully resolve the ancestral nodes of the tree, we investigated the divergence of protein domains in the V-H^+^PPase molecules. Using Protein family (Pfam) database, we found a domain in the protein, PF03030. The domain is shared by 31 species in Eukarya, 231 in Bacteria, and 17 in Archaea. The universal distribution of the V-H^+^PPase PF03030 domain, which is associated with the V-H^+^PPase function, suggests the domain and the enzyme were already present in the Last Universal Common Ancestor (LUCA).

**Conclusion:**

The importance of the V-H^+^PPase function and the evolutionary dynamics of these domains support the early origin of the acidocalcisome organelle. In particular, the universality of volutin granules and presence of a functional V-H^+^PPase domain in the three superkingdoms of life reveals that the acidocalcisomes may have appeared earlier than the divergence of the superkingdoms. This result is remarkable and highlights the possibility that a high degree of cellular compartmentalization could already have been present in the LUCA.

**Reviewers:**

This article was reviewed by Anthony Poole, Lakshminarayan Iyer and Daniel Kahn

## Background

According to the theory of serial endosymbiosis [1], the symbiotic history of mitochondria and plastids started more than 1.5 billion years ago when a primitive eukaryotic cell engulfed a bacterium [2]. These endosymbionts gave rise to contemporary organelles through complex, and poorly understood molecular evolutionary events. It is now widely accepted that an α-proteobacterium was the ancestor of mitochondria [3]. Similarly, a single endosymbiotic association between a cyanobacterium and a mitochondriate eukaryote has been proposed as the origin of the chloroplast [2]. Although the endosymbiotic origin of plastids is well established [4,5], the events that drove the evolution of other sophisticated membrane-bound cellular compartments remain unclear. In fact, other mechanisms may be responsible for the generation of organelles.

Prokaryotic cells have been simplistically portrayed in the general scientific literature as a plasma membrane "sack" containing DNA, filled with cytoplasm and surrounded by a cell wall. However, provocative findings have challenged the notion that the prokaryotic cell lacks a sophisticated cytoplasmic organization. For years, it was believed that the shape of bacterial cell was maintained by the organization of cellulose fibers within the cell wall. However, a protein similar to actin, which was once thought to be exclusive to the eukaryotic cytoskeleton, was discovered in bacteria [6-8]. This protein called MreB self-assembles into filaments forming a cytoskeleton-like structure that maintains the shape of bacterial cells.

Even more recently, the idea that organelles similar to those present in eukaryotes are absent in bacteria was challenged by the discovery of an organelle similar to the acidocalcisome of unicellular eukaryotes within the bacterium *Agrobacterium tumefaciens *[9]. The existence of this bacterial organelle has also been confirmed in the photosynthetic bacterium *Rhodospirillum rubrum *[10]. The acidocalcisome is an acidic calcium-storage organelle that was first described in trypanosomes, but has now been found in the cells of diverse organisms ranging from bacteria to higher eukaryotes [11]. This membrane-enclosed organelle is characterized by its acidic nature, high electron density, and high content of polyphosphates (polyP) including pyrophosphate (PP_i_), calcium, magnesium, and other elements. In addition, the organelle contains a variety of cation pumps including Ca^2+^/H^+ ^and H^+ ^pumps. In particular, the vacuolar proton translocating pyrophosphatase (V-H^+^PPase) proteins have been localized in the acidocalcisomes of bacteria, parasitic protozoans, algae, plants, and recently in cockroaches [12]. The widespread distribution of these proteins, which also contain ancient, highly conserved protein motifs, suggests that V-H^+^PPase arose early in the evolution of life on Earth. As mentioned before, the main components of the acidocalcisome are polyphosphates, which includes PP_i_. V-H^+^PPases use PP_i _as an energy source to pump protons into the acidocalcisome lumen, thereby generating an electrochemical gradient. This strongly suggests that both the V-H^+^PPase and the acidocalcisome share similar evolutionary histories.

Acidocalcisomes are morphologically and chemically similar to the structures historically described as volutin or polyphosphate bodies, which have been identified in a variety of microorganisms, including bacteria, archaea, algae, and protozoans [13]. Meyer [14] described volutin granules in bacteria more than 100 years ago and much later they were identified as acidocalcisomes [15]. In protists, this organelle has essential roles in the regulation of intracellular Ca^2+^, pH, and osmotic homeostasis, in which polyP is a key player. However, the study of polyP has been neglected for years. An important feature of polyP is its negative charge, which enables it to interact with nucleic acids and act as a regulator of RNA polymerases [16] and proteinase activity [17]. In addition, these negatively charged polymers can serve as a binding template for proteins, amino acids, polysaccharides and many cations [18-20].

Other membranous structures have also been reported in bacteria as well [21]. However, these organelles are not found in the eukaryotes (e.g., magnetosomes of magnetotactic bacteria [22]). Thus, the acidocalcisome is the first and the only organelle demonstrated to be present in both bacteria and eukaryotes, and its morphological, structural and ultrastructural, chemical and biochemical, and molecular links to volutin granules may suggest it is indeed universal. The conservation of this organelle in prokaryotes and eukaryotes suggests that it has important functions that still await discovery [11].

Debates about organellar evolution have been revitalized and streamlined during the post-genomic revolution. The availability of multiple complete genome sequences from the whole spectrum of life, along with corresponding protein sequence data sets and more sophisticated bioinformatics tools opens a window into the intricate mechanisms of cellular evolution. In the present study, we use sequences and domain information from a diagnostic protein of the acidocalcisome, the proton pump V-H^+^PPases, to infer the phylogenetic history of this protein and an ancient origin of the acidocalcisome. Within protein molecules, certain parts can interact more strongly with each other than with other parts, and are usually highly conserved, making up modules, called protein domains [23]. These segments fold compactly, appear repeatedly in different proteins, and often combine with other domains. Because domains can harbor functional centers or can modulate biological activities, the combination of domains in proteins adds inherent diversity to the biological make up of an organism [24]. The combination of domains in proteins can help clarify the origin and the evolutionary histories of proteins and proteomes [25]. Using Bayesian reconstruction methods, we build phylogenetic trees base on protein sequences that shed light on the evolution of V-H^+^PPase proteins. We then examine the distribution of the main functional V-H^+^PPase domain on the tree using Protein family (Pfam) database definitions.

Here, we delineate the connections between the evolution of V-H^+^PPases and volutin granules as opportunity to infer the likely origin of the acidocalcisome. In addition, we discuss the implications that these findings may have on our understanding of the cellular complexity of the ancestor of cellular life.

## Results and Discussion

### Taxonomic distribution of V-H^+^PPase pumps

V-H^+^PPase is commonly present in the membrane of acidocalcisomes which couple PP_i _hydrolysis to active proton transport across the organellar membrane. The enzyme is found in organisms from all superkingdoms of life and should be considered ancient. Its ubiquitous nature already suggests it appeared before the last universal common ancestor (LUCA) of diversified life [26]. The V-H^+^PPase is a highly conserved enzyme that acidifies the lumen of the acidocalcisome [11]. V-H^+^PPase has been detected by immunogold labeling localization in acidocalcisomes of bacteria, and several eukaryotic microorganisms [9,10,27-33]. Recently, an organelle that shares similar characteristics of the acidocalcisome, including the presence of V-H^+^PPases in its surrounding membrane, was found inside the protein storage vacuole of seeds [34]. Additionally, an acidocalcisome-like organelle with membrane-bound V-H^+^PPases has been identified in the egg yolk of cockroaches [12]. These studies show that a bona fide organelle linked to V-H^+^PPase function exists in bacteria, microbial eukaryotes, and higher eukaryotes.

### Volutin granules: A universal phenomenon

**Figure 1:**
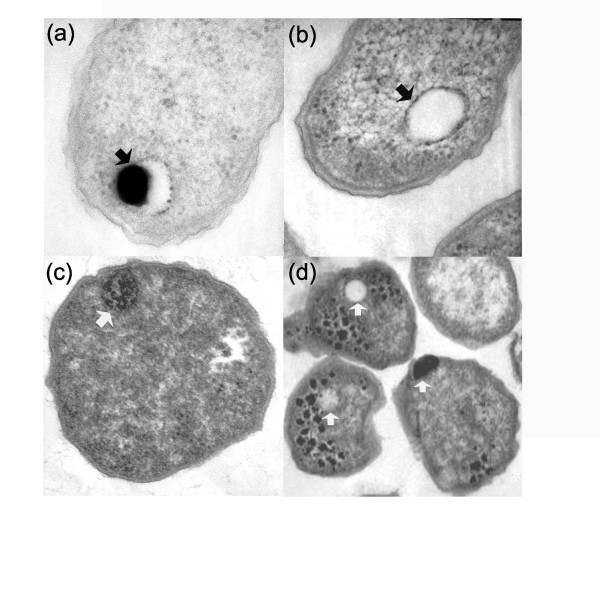
**Electron micrographs thin sections of *Agrobacterium tumefaciens(a &b) *and *Methanosarcina acetivorans *(c & d)**. In panel (**a**), the arrow shows the partially filled acidocalcisome of *A. tumefaciens *containing electron dense material. In panel (**b**), the arrow shows an empty *A. tumefaciens *acidocalcisome. In panel (**c**), the arrow shows the electron dense volutin granule of *M. acetivorans*. In panel (**d**), the arrows show empty, partially, and completely filled volutin granules of *M. acetivorans*.

Volutin-polyP bodies occur in organisms spanning an enormous range of phylogenetic complexity from Bacteria and Archaea to unicellular eukaryotes to algae to plants to insects to humans. Volutin/polyphosphate electron dense granules exhibit varied internal patterns suggestive of sponge-like electron-dense spheres, and when fixed they look as empty or partially empty vacuoles. The volutin granules shown in a number of microorganisms appear to be identical to acidocalcisomes of *Agrobacterium *and *Rhodospirillum *and eukaryotes. While it may come as a surprise that volutin electron-dense granule from different organisms bear close similarity to acidocalcisomes, now we know that acidocalcisomes are virtually identical in size, composition and morphology to volutin-polyP bodies found in a vast array of organisms, including Archaea. Although the volutin granules present in *Methanosarcina *have not been yet confirmed to be acidocalcisomes, they are morphologically and chemically similar to the acidocalcisomes of *Agrobacterium and Rhodospirillum *[9,10]. The volutin granules of *Methanosarcina *have the same chemical profile, morphological characteristics (sponge like-structure) and high levels of phosphorous compounds and calcium [35-37] than acidocalcisomes. In addition, some of the *Agrobacterium *acidocalcisomes (Figure 1, panels A and B) appear like partially or empty vacuoles due to the fixation/staining protocol that may promote the diffusion of the electron dense material out of the acidocalcisome. Remarkably, the same phenomenon is observed in the volutin granules of *Methanosarcina *(Figure 1, panels C and D).

Therefore, volutin-polyphosphate bodies electron dense granules are distributed throughout phylogeny and are universal. Consequently, our results support the hypotheses that the volutin granules of Archaea are likely acidocalcisome structures and that acidocalcisomes are universally distributed.

### Phylogenetic analysis of V-H^+^PPase

**Figure 2:**
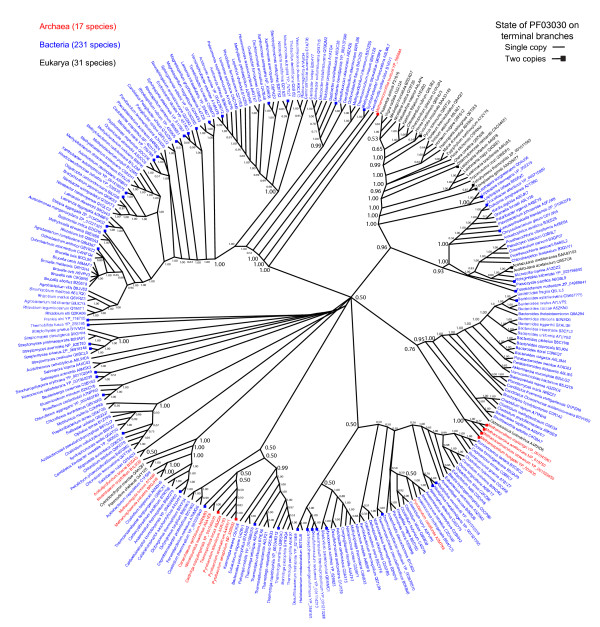
**Unrooted phylogram from Bayesian analysis of 279 V-H**^**+**^**PPase sequences from the three superkingdoms of life**. Terminal branches of V-H^+^PPases are labeled with a solid square when the Pfam domain was found duplicated in the sequence. If the domain were found single, the terminal branch has no square. All the branches are labeled with the name of the species and accession number. Eukaryote species are represented with black letters, Bacteria with blue and Archaea with red.

The tree of V-H^+^PPase amino acid sequences is star-like and without much deep internal topological structure, suggesting the existence of limited phylogenetic signal in the sequence needed to dissect deeper phylogenetic relationships (Figure 2). It also suggests a relatively recent history of sequence diversification in the molecule. The eukaryal, bacterial and archaeal V-H^+^PPase sequences largely formed monophyletic groups and eukaryotic sequences mostly grouped according to established organismal classification, with unicellular eukaryotes being placed at the base of the clade and unicellular algae and plants diversifying later. However, there were several instances of sequences that group in a way contradictory to accepted classification. These atypical patterns could be explained by lateral gene transfer (LGT) events among Bacteria, Archaea and Eukarya, some of which may have occurred early in evolution. Alternatively, these unusual placements might simply be the result of phylogenetic error associated with using only a single gene for analysis [38].

The fact that V-H^+^PPase sequences group many familiar clades monophyletically, and are also ubiquitous among superkingdoms although with poor phylogenetic signal, suggests these proteins are truly ancient. To further explore their ancestral nature we focused on structure, which is highly conserved in evolution [39]. We therefore, identified protein domains in the V-H^+^PPase sequences, mapped them onto the V-H^+^PPase tree, and determined how many species in the three superkingdoms harbored these domains.

### Evolution of Pfam domains in V-H^+^PPase proteins

V-H^+^PPase proteins have not been crystallized, and there is no structural entry associated with them in the Protein Data Bank (PDB). This absence probably stems from the limitation of NMR and X-ray crystallographic techniques in their ability to acquire high-resolution structures from proteins associated with membranes [40]. In fact, only 1% of structural PDB entries are membrane proteins. We nevertheless considered the possibility that some other known structure could match the structure of V-H^+^PPase. However, domain search using Hidden Markov Models (HMMs) of fold superfamily and fold family structural recognition revealed no hits. It has already been established that V-H^+^PPase proteins have domains with sequence motifs that are highly conserved in evolution [41], which suggests that catalytic domains harboring the motifs in V-H^+^PPase are very ancient. Because Pfam attempts to classify protein domains and families using sequence alignments and profile HMM analysis of sequence motifs [42], we explored the distribution of Pfam domains in the sequences of V-H^+^PPase proteins. A Pfam search revealed that all V-H^+^PPase sequences have a single domain that is annotated as inorganic H^+ ^pyrophosphatase, PF03030. This domain in our analysis is distributed in 279 species including Eukarya, Bacteria, and Archaea (Figure 2). The domain PF03030 has a conserved motif (57 amino acids) that contains a functional active site for the V-H^+^PPase (Additional File 1). It has already been established that a consensus sequence of the motif is highly conserved across more than three hundred V-H^+^PPase protein sequences obtained from completed genomes and Sargasso Sea metagenomic sequences [41]. Our data indicate that the domain PF03030 is conserved in all three superkingdoms, supporting our hypothesis that the protein appeared before the LUCA. The taxonomic incongruences present in Figure 2 are clearly the result of secondary evolutionary events of LGT that are derived or simply arise from poor phylogenetic signal in V-H^+^PPase sequence.

### Origin of the acidocalcisome

Several hypotheses attempt to answer the question of when and how the acidocalcisome arose. The canonical explanation that the acidocalcisome was acquired either through lateral transfer or via an endosymbiotic event is, however, unlikely. Furthermore, sequence and domain analysis of the V-H^+^PPase and the universal distribution of volutin granules support the appearance of the volutin granules and possibly the acidocalcisome before the initial branching of the tree of life (Figure 2). Neither LGT nor direct filiation from the α-proteobacteria appears to be a likely explanation for the organelle's acquisition in eukaryotes. Instead, the most parsimonious explanation would be to posit that the volutin granule/acidocalcisome appeared very early, before the branching of the three superkingdoms of life, i.e., that the volutin granule/acidocalcisome was likely present in the LUCA. Because acidocalcisomes have been confirmed in Eukarya and Bacteria, and volutin granules that are remarkably similar to acidocalcisome like-structures have been observed in Archaea [35-37], these findings support the view that this organelle might have been conserved in all superkingdoms. Even if volutin granules of Archaea are not confirmed to be genuine acidocalcisomes and represent ancient forms of the acidocalcisomes organelle, the universal presence of the organelle precursor (volutin granules) suggests that the LUCA had a more complex cytoplasm organization than previously thought. Moreover, the PF03030 domain which carries the V-H^+^PPase proton pumping molecular function crucial for the acidification of the acidocalcisome using PP_i _as substrate (see below) is distributed universally. For these reasons, we hypothesize that this organelle or its precursors were very early cellular innovations that occurred prior to major organismal lineage diversification events. As the lineages of the tree of life began to diverge, this organelle or its precursors were inherited in the three domains by direct descent from the LUCA. Under this scenario, the putative absence of the organelle in an organism must be explained as a loss.

It is outside of the scope of the present work to discuss the characteristics of the LUCA, but the above hypothesis implies that the ancestral lineage may have had a high degree of cellular sophistication, as others have suggested [43-46]. For example, Collins [47] argued that introns and spliceosomal machinery in the eukaryotic ancestor might have been similar in overall complexity to that seen today. Likewise, protein architecture offers another clue because a substantial fraction of domain structures in the protein world are common to all organismal superkingdoms and are truly ancient [23].

While the 3D structure of the proton V-H^+^PPase pumps is not yet available, a recent analysis revealed that sequences from this protein family has a very ancient and strongly conserved 57-residue block with unique properties [41]. This finding also supports the idea that V-H^+^PPases appeared very early in evolution of life. Our analysis of the distribution of the main Pfam domain along the V-H^+^PPase phylogenetic tree reinforces this hypothesis (Figure 2). In addition, one of the main functions of the acidocalcisome, the modulation of polyP homeostasis, could offer further insight into its evolutionary link with V-H^+^PPases. The most striking feature of acidocalcisome is the capacity to store polyP and PPi. This polymer is present in every living cell, and was long thought to be a molecular fossil conserved from a prebiotic time with no remaining cellular function in the cytoplasm of cells. However, this molecule has been rediscovered, and evidence is mounting for the many functions of polyP. PolyP is an anionic, negatively charged molecule. Its length varies from the dimeric pyrophosphate to chains of thousands of orthophosphate residues linked by phosphoanhydride bonds, similar to those found in ATP. PolyP is a molecule that was apparently present on Earth before life appeared [48,49]. When the planet was still hot, phosphate might have occurred largely in the form of polyP, which was formed by a wide variety of abiotic processes including the dehydration of orthophosphate at high temperatures [50]. However, it was recently proposed that the prebiotic chemistry of phosphorus was shaped by phosphorous (P) from extraterrestrial material, called schreibersite, (Fe,Ni)_3_P. This mineral reacts with water to form more soluble reduced P compounds; thus, the oxidation of schreibersite in water forms several potentially prebiotic phosphorous species including phosphite, pyrophosphate and triphosphate, and phosphonates [51]. Alternatively, it has been suggested that in the early Earth, cyanate might have mediated the synthesis of PP_i _from CaHPO_4_·2H_2_O (brushite) [52]. Brushite is the only solid species formed between pH 6 and 7, which is thought to have been in the pH range of the earlier marine environment [53].

PP_i _and polyP can be used as a source of energy. However, the developments of proton gradients are a more sustainable strategy to generate potential energy available for work in a cell. Proton gradients are as universal as proteins and nucleic acids in living organisms. Therefore, the acidocalcisome, acting like an energy source, might have played a critical function for survival of the LUCA cells. Interestingly, V-H^+^ATPases and ATPsynthases, which are capable of creating proton gradients across the cell membranes, are among the most ancient protein structures that are known [23]. In addition, it has been suggested that the catalytic and noncatalytic subunits found in these proteins evolved from the same enzyme already present in the LUCA [54-56]. Remarkably, the V-H^+^ATPase was found in the acidocalcisome of several eukaryotic species together with the V-H^+^PPase [11,57]. Both V-H^+^PPase and V-H^+^ATPase couple the transport of protons into cellular compartments and enhance the acidification of the lumen of this organelle [11]. Moreover, V-H^+^ATPases can "run in reverse" to synthesize ATP when subjected to a sufficiently large H^+ ^electrochemical gradient [55], while V-H^+^PPsaes can have the same ability to regenerate pyrophosphate[58,59]. PP_i _can be used as an alternative to ATP, because of its similarity to the ATP bond structure. In addition, PP_I _acts as a biological energy donor in photosynthesis [60,61]. Therefore, it has been suggested that PP_i _might have been involved as an energy carrier during the early evolution of life [62]. Taken together, these observations suggest that the LUCA might have used the polyP stored in the acidocalcisome as a substrate for its V-H^+^PPases, which together with the V-H^+^ATPase make the acidocalcisome capable of sustained energy generation. As a result, the energy demands of the LUCA in the harsh environments of early Earth might have been met by a combination of polyP hydrolysis, creation of a H^+ ^gradient, and regeneration of ATP and PP_i_. Moreover, polyP may have facilitated the assembly and orientation of the key molecules such as phospholipids, nucleic acids, and proteins [63]. The multifunctionality of polyP makes this molecule a unique link between living organisms and the inorganic world. PolyP structural and physico-chemical characteristics are likely the reason this molecule was selected as a core component of living cells during evolution [18]. Although the chemical identity of polyP has remained unchanged, its function might have become more specialized during the organism diversification from the ancestral cells to apparently simple bacterial cells and the platelets of mammals. Now a large body of emerging evidence has begun to elucidate the role of polyP in a wide variety of physiological processes including physical and chemical stresses. Indeed, one of the main functions of the acidocalcisome is linked to cellular responses to environmental stresses [64]. Therefore, it would be entirely plausible to hypothesize that the presence of the acidocalcisome in the LUCA might have been advantageous for adapting to the environment of the early Earth.

## Conclusions

The sequence and domain analysis of the V-H^+^PPases together with the characteristics and universal distribution of the volutin granules and its main component, polyP, offer pertinent clues about the evolutionary history of the acidocalcisome. Our findings suggest that the V-H^+^PPases, which are universally distributed and tightly associated with the acidocalcisome, originated prior to the divergence of the three superkingdoms. Interestingly, volutin granules-polyphosphate bodies that have been detected in *Methanosarcina acetivorans *and other Archaea [35-37] are remarkably similar to acidocalcisomes of *Agrobacterium *and *Rhodospirillum *[9,10]. Confirmation of the existence of the acidocalcisome in the Archaea using biochemical, enzymological and cytological characterization will be significant because it will further support the hypothesis that this organelle or its precursors were indeed inherited directly from the LUCA. Further characterization of the prokaryotic acidocalcisome may confirm the presence of other proteins previously detected within the eukaryotic acidocalcisome. These findings could be used for a more comprehensive analysis of the evolutionary history of the acidocalcisome. We trust these results will shed new light in understanding the putative complexity of the LUCA and the early evolution of the three superkingdoms of life.

## Methods

### Sequences and phylogenetic analysis

Sequences for vacuolar pyrophosphatases were downloaded in FASTA format obtained from the Pfam database (see below), and were aligned with Clustal W [65]. Ambiguously aligned sites were removed using BioEdit ver. 7.0.9 [66].

We initially collected 98 V-H^+^PPase sequences from organisms spanning the three superkingdoms of life including the unicellular parasites *Trypanosoma*, *Toxoplasma *and *Plasmodium *[26,27] and the green algae *Chlamydomonas reinhardtii *[28], where acidocalcisomes were first identified. The Pfam search indicated that all of the 98 sequences have one or two PF03030 (V-H^+^PPase) domain. Consequently, we retrieved all the protein sequences that harbor the domain from the Pfam database. As a result, 776 protein sequences in total were obtained and then aligned and edited, and imported into phylogenetic reconstruction using Bayesian MCMC methods. By manually examining the topology of the phylogenetic tree, we removed duplicated sequences with no genetic distance and sequences that are not identified taxonomically. Subsequently, sequences that have paralogous relationships in the tree were totally removed in this analysis because it is difficult to know which sequences of paralogs are true orthologs for the pyrophosphatase on the tree. We finally selected 279 out of 776 pyrophosphatase sequences that consist of 31 eukaryal, 231 bacterial and 17 archaeal species (Additional File 2). The multiple sequence alignment for the sequences followed by deletion of ambiguously aligned sites resulted in 1,652 sites. The conservation scores of individual aligned sites were calculated using JALVIEW version 2 [67].

From the multiple sequence alignments manually edited, phylogenetic trees were reconstructed using MrBayes version 3.1.2 [68], which runs two independent Markov chain Monte Carlo (MCMC) chains for each analysis and checks for convergence between them. The JTT [69] model of amino acid replacement was used, and 4 chains were run for each analysis, sampling trees every 100 generations. 2,000,000 generations were run, and 5,000 trees (25%) were discarded like burnin. The resulting tree files were summarized into a consensus tree showing posterior probabilities, visualized, and color-coded using Dendroscope version 2 [70]. We excluded the aligned sites that include gaps in the multiple sequence alignment and calculated the overall mean distance among the 279 sequences of the alignment using MEGA5 with the JTT model [71]. For each of the non-aligned 279 sequences, the topology of transmembrane helices was predicted using SCAMP with default parameters [72]. We also used several other algorithmic implementations, including TMMOD, an HMM-based transmembrane protein prediction implementation [73], and TOPCONS, a consensus prediction tool of membrane protein topology [74].

### Protein domain analysis

We used the Pfam database version 24.0 to assign protein domains to the vacuolar pyrophosphatase sequences [42]. On the Pfam website, http://pfam.sanger.ac.uk/family/PF03030#tabview=tab6, we performed a batch search for the sequences against both the Pfam-A HMMs using an E-value of 10^-4 ^[75]. To assign protein domains at the levels of fold superfamily and fold family that are defined in the Structural Classification of Proteins [76], we downloaded a MYSQL database from the SUPERFAMILY database, version 1.73 [77] and searched the sequences against HMMs of fold superfamilies and fold families using the iterative sequence alignment and modeling system (SAM) method [78]. As with the Pfam search, an E-value of 10^-4 ^was used because it represents an ideal cutoff to minimize false positive assignments in the HMM searches [79].

### Electron Microscopy

*Agrobacterium tumefaciens *cells were washed with Dulbecco's PBS and fixed for 1 h with 2.5% grade II glutaraldehyde, 4% freshly prepared formaldehyde, 0.03% CaCl_2_, and 0.03% picric acid in 0.1 m cacodylate buffer, pH 7.2. Bacteria were post-fixed with OsO_4 _for 45 min and then for 15 min with potassium ferricyanide, washed, and treated with 2% uranyl acetate for 30 min. Subsequently, samples were dehydrated by successive incubations of 6 min with increasing concentrations of ethanol (10, 25, 36, 75, 95, and 100%) at room temperature. Epoxy embedding was carried out by resuspending the sample once in 1:1 ethanol/acetonitrile, twice in 100% acetonitrile, and then 30 min in 1:1 epoxy/acetonitrile, 1.5 h in 3:1 epoxy/acetonitrile, and 4 h in 100% epoxy. Embedded samples were polymerized for 20 h at 85°C. Epoxy blocks were ultrathin-sectioned, sections were deposited on 300-mesh copper grids and grids were stained with uranyl acetate for 30 min and triple lead stain (lead citrate, lead nitrate, and lead acetate) for 1 min. For *Methanosarcina acetivorans*, cells were processed for electron microscopy as described in [80].

## Abbreviations

V-H^+^PPases: Vacuolar proton-translocating pyrophosphatases; Pfam: Protein family; LUCA: Last universal common ancestor; LGT: Lateral gene transfer; PDB: Protein Data Bank; HMMs: Hidden Markov Models; SAM: Sequence alignment and modeling system

## Competing interest statement

The authors declare that they have no competing interests.

## Authors' contributions

MJS, JW and GCA conceived and designed the experiments. MJS, JW, GCA, KMK and AV performed the experiments and/or participated in data analysis.

MJS, JW, GCA, and KMK wrote the paper. All authors read, edited and approved the final manuscript.

## Reviewers' comments

### Reviewer's report 1

Anthony Poole (University of Canterbury, New Zealand)

The work presented here makes an interesting case for the existence of compartmentation early in evolution, based on the presence of volutin granules/acidocalcisomes in all three domains of life. The authors present data indicating that vacuolar pyrophosphatases are found in all three domains of life, and interpret this as consistent with their conjecture that acidocalcisomes predate the divergence of the three domains. It is clear that the phylogeny of vacuolar pyrophosphatases cannot be used to strongly argue for presence of this gene in the LUCA, partly because of poor resolution in the dataset, and partly because of unambiguous evidence for horizontal gene transfer within the dataset. While there are some clear cases of late transfers in their phylogeny, it is harder to establish events deeper in the tree. Mapping the domain architecture of PF03030 onto this tree does not obviously strengthen the evidence, though it is striking to note the high level of conservation of the domain itself.

While it certainly seems reasonable to expect that a complex organelle may only have arisen once in evolution (meaning acidocalcisomes should be considered ancient), such expectation is not evidence in itself. In further testing this conjecture, it will therefore be interesting to identify a broader set of common signature proteins associated with this organelle, and examine their evolutionary histories. In addition, examining whether patterns of gain and loss of both the organelles and key associated proteins can be ascertained using more detailed phylogenies and character analyses within a phylogenetic framework (as was successfully demonstrated for hydrogenosomes - see Embley et al. Phil. Trans. R. Soc. Lond. B (2003) 358, 191-203) will provide an important means of testing the conjecture presented here.

The following major claims are made on the basis of the data presented:

1. That Archaea possess membrane-bound acidocalcisomes/volutin granules.

2. That volutin granules evolved prior to the divergence of the three domains.

These are bold claims. However, my reading of the manuscript leads me to conclude that the authors do not currently have sufficient evidence to fully support either of these claims. I address each in turn.

1. That Archaea possess membrane-bound acidocalcisomes/volutin granules.

Figure 1 presents electron micrographs of *Methanosarcina acetivorans *cells (panels c & d) wherein intracellular structures are clearly visible as either black (electron dense) or white (putatively empty). The resolution of panels c & d make it difficult to see any evidence for a membrane; plate d in particular is pixellated. I therefore find it difficult to evaluate the presented evidence as being either supportive or dismissive of a membrane surrounding these structures. No other cell biological evidence is given to support the interpretation presented in the paper, in notable contrast to the careful and extensive experimental evidence previously presented by the lead author for the characterization of acidocalcisome structures in the bacterium *Agrobacterium tumefaciens *(J Biol Chem 278, 29971-8). The results in figure 1 therefore are far too preliminary to be presented in support of acidocalcisomes or acidocalcisome-like structures in Methanosarcina.

I note that proton pyrophosphatase pumps have previously been identified in Archaea, having been experimentally characterized in *Pyrobaculum aerophilum *(Drozdowicz et al. 1999 FEBS Lett 460:505-12) -- it seems somewhat surprising that no reference to this paper is made. From what I can ascertain, no data indicate an association of these pumps with any internal membrane structure in *Pyrobaculum*. I am therefore curious as to whether acidocalcisome-like structures have been characterized in this archaeon. Finally, given that those authors were able to express the *Pyrobaculum *pyrophosphatase in yeast, have antibodies have been successfully raised against this enzyme? This is helpful information, since my reading of the literature indicates that these pumps are known to associate with acidocalcisomes, though not exclusively; with that in mind, it is rather a leap to equate presence of H+-PPases in Archaea with the presence of acidocalcisomes. Experiments of the sort presented in the above-noted JBC paper are essential for any claim for the existence of such structures in Archaea.

*Authors' Response: In this paper we neither directly claim that the volutin granules of Archaea are acidocalcisomes nor do we explicitly extrapolate acidocalcisome presence based on identification of V-H^+^-PPases in Genbank. What we are postulating is that the volutin granules observed with electron microscopy in Methanosarcina are indeed very similar to the volutin granules of Agrobacterium and Rhodospirillum, which now are known to be acidocalcisomes. In addition, our hypothesis is based on previous publications where micrographs of the volutin granules of Methanosarcina and other Archaea such as Acidithiobacillus ferrooxidans and Sulfolobus metallicus are structurally very similar to the acidocalcisomes found in bacteria. Our intention of showing these structures is to provide a visual connection between volutin granules (which we do have direct evidence of) and acidocalcisomes (an inference that requires considerably more evidence to demonstrate)*.

*We consider that the information we present in this paper is significant because it provides concrete facts and clues about the possible presence of this organelle in Archaea. In addition, this work prompts further inquiry and exploration of this organelle in other organisms (including the full characterization of the volutin granules in Archaea as suggested by the reviewer). The full characterization of the volutin granules of Methanosarcina is not a trivial matter. Based on similar previous studies, it might well take 1-2 years of work to complete. Therefore, the present scope of this paper is not to prove that the volutin granules of Methanosarcina are acidocalcisomes, but instead illustrate our hypothesis that they are promising candidates (among others) to be acidocalcisomes. We appreciate the note about the work of Drozdowicz et al. (1999). The reference has now been included. Originally, we did not include this reference because the discovery of V-H^+^PPase in Pyrobaculum was made more than 10 years ago and we considered this information to be common knowledge (we did include this species in our analysis). Interestingly, Drozdowicz et al. (1999) also made an early suggestion of the possibility that the V-H^+^PPases could have originated in the last universal common ancestor. However, and as the reviewer points out, no data is presented in the paper that indicates an association of these pumps with any internal membrane structure in Pyrobaculum. This is not surprising. At the time of the publication of this paper, the association between volutin granules and acidocalcisomes in bacteria had not been reported. Moreover, the absence of organelles (similar to eukaryotic organelles) in bacteria was at that time already considered a dogma in microbiology. Consequently, the search for a membrane bound organelle in bacteria was not undertaken. In the case of Agrobacterium for example, the presence of volutin granules and vacuolar pyrophosphatases were described a long time ago, but it was not until 2003 when the volutin granules of Agrobacterium were rediscovered as acidocalcisomes and the V-H^+^PPase was located in their surrounding membrane. Even more eloquent is the fact that in Rhodospirillum rubrum, the microbe where the V-H^+^PPase was first discovered, the volutin granules were never investigated until 2004, when they were confirmed as acidocalcisomes and the V-H^+^PPase also localized in their surrounding membranes*.

2. That volutin granules evolved prior to the divergence of the three domains.

The authors also present genomic evidence (H+-PPase sequences) in support of acidocalcisomes in archaea. The implicit argument in this part of the paper is that one can extrapolate acidocalcisome presence based on identification of H+-PPases in Genbank.

There are two interesting points here. First, it is nice to see that the authors are honest regarding the limits of phylogeny in this particular instance. The previously inflammatory acronym MUTOG (Myth of the Universal Tree from One Gene -- coined by David Penny) does need to be taken seriously following the recognition that most genes do not carry sufficient phylogenetic signal to trace the relationships between species across the three domains. Moreover, with HGT an indisputable factor in gene distribution, this caution is not only welcome, but necessary. Against this backdrop, that a clearly resolved three-domain tree cannot be generated for the V-H^+^PPases (for instance, the archaeal sequences do not form a monophyly: *Methanococcoides *is on alone branch, *Pyrobaculum *and *Caldivirga *form a clade, and there is a third clade of five sequences, including *Methanosarcina acetivorans*, that connects the tree at the large central polytomy) is not evidence against an ancient pre-LUCA origin. Having said that, it is not evidence in favor of it either. The dual forces of mutation and gene transfer are having a major impact on the reconstruction of the deep evolutionary past and it may be that signal has in most cases been heavily degraded, to the point that it cannot be informatively identified.

The second point lies at the heart of this paper. The triumph of endosymbiotic theory has been the recognition that some key eukaryotic organelles did not evolve de novo, but are instead the result of one cell becoming resident within another. The emerging view of complex membrane structures has therefore been one of continuity (TCS), though this may yet be challenged by the recognition that both bacteria and archaea can harbor complex cell structures. In the case of acidocalcisome/volutin granule organelles, it seems plausible to suggest a deep evolutionary origin, given the presence of both the H^+^PPases and these organelles across representatives from all three domains of life. This would suggest that multiple losses have occurred throughout evolution, from a more compartmentally complex common ancestor. What we still don't know, and are challenged to think about by this paper, is whether complex internal membrane structures such as these could arise multiple times independently. In this respect, comparative analyses do have an important role to play in establishing what the minimum number of components required to support an acidocalcisome or volutin granule may be. The variability in membrane number and architecture observed across Planctomycetes (Fuerst) does suggest a greater capacity for evolution of novel membranes than suggested by a stringently 'genetic' view (sensu Cavalier-Smith) of membrane evolution. More fine-scale examination of whether the distribution of key proteins localized to acidocalcisomes/volutin granules completely overlaps with the existence of these organelles. In addition, looking at evolution within a group (e.g. relatives of *Agrobacterium tumefaciens*) may help elucidate whether or not horizontal gene transfer does play a role in the emergence of such complex cell structure. It might be time to ask just whether our assumptions about how 'hard' it is to evolve a compartment are in need of scrutiny.

*Authors' Response: We agree with the reviewer that the poor phylogenetic resolution of the tree generated from V-H^+^PPase gene sequences, a common finding in many genes that exist in a genome, tells little about the ancestrality of the V-H^+^PPase gene. Lack of phylogenetic signal in the V-H^+^PPase sequences is probably the result of (i) high sequence conservation of segments that correspond to the Pfam 03030 domain that are present in each and every one of the sequences examined, including sites necessary to maintain fold structure and sites needed for catalytic activity, and (ii) segments of sequence that are highly variable and prone to sequence saturation that contain highly degraded phylogenetic signal and probably arise from the complex and frustrated interplay between stability and function that exists in proteins *[81]*. In contrast, the universal distribution of the Pfam 03030 domain in the tree of V-H^+^PPase sequences and in every other V-H^+^PPase sequence we examined provides strong support to the suggestion that the functional and structural core of this domain was already present in LUCA and was vertically distributed without loss in all lineages of the tree of life. The size of the domain (an average of 603 amino acids covering 94% of the sequence with a model length of 682 amino acids) was calculated based on start and end positions on the HMM (Pfam) alignment of sequences obtained by searches in the Sanger database. The average identity of the complete alignment is 44%. Despite sequence variability, the domain is therefore highly conserved and universally present. More information about seed sequences and PF03030 domain distributions can be found elsewhere. (http://pfam.sanger.ac.uk/family/PF03030#tabview=tab6).*

*The second point raised by the reviewer is important for the genesis of organelles and cellular structure. The proteome of LUCA was recently reconstructed and was shown to embody the functions of a complex organism, with pathways for the biosynthesis of membrane sn1,2 glycerol ester and ether lipids *[82]*. An ancient volutin/acidocalcisome structure present in LUCA implies gradual evolution of internal cellular structure in the universal ancestor of cellular life. By definition, LUCA implies the absence of cellular lineages. Consequently, a process of endosymbiosis between lineages similar to that used to explain the origin of mitochondria or plastids could not be responsible for the rise of volutin/acidocalcisome organelles without invoking endosymbiosis was an ancient and pervasive property of LUCA and that these polyphosphate harboring cellular structures were remnants of that cellular state. Alternatively, cellular structure other than an external membrane may be the logical consequence of energetic and architectural constraints operating on membrane organization *[83]*. In vitro experiments of membrane biogenesis show considerable diversity of cellular shape and structure, with cellular engulfment being a common outcome *[84,85].

The argument presented here, that compartmentation is much older than we may expect based on the presumed late origin of the eukaryote nucleus and endomembrane system, has been given an additional boost in this manuscript from Seufferheld and colleagues. However, the jury is certainly still out -- on the present data, it is difficult to distinguish between either a deep evolutionary origin with numerous losses, or 'convergent' origins (of the organelles, not the H^+^PPases). We do not yet know whether independent recruitment (via horizontal transfer) of key functionalities, such as the H^+^PPases, are sufficient for the independent evolution of a new membrane-bound organelle. The authors make the reasonable assumption that loss is more likely, and bolster their argument by relating their provocative model a possible early role for pyrophosphate/polyphosphate in the origin and early evolution of life. The obvious problem with scenarios that invoke loss from a more complex ancestral state is that one also needs to explain the original gain. The present manuscript does not give us an answer, but this and other results do challenge us to keep an open mind.

Authors' Response: A scenario of multiple parallel or convergent origins of volutin/acidocalcisome cellular structures in lineages is possible but is less parsimonious than a single origin in LUCA. The reviewer however is right that a more comprehensive analysis of molecular machinery responsible for key functionalities of this compartment will help understand the role that horizontal gene transfer and molecular recruitment may have in shaping cellular structure. Phylogenomic analysis of the acidocalcisome-associated protein repertoire could be very informative in this regard.

### Reviewer's report 2

Lakshminarayan Iyer (NIH/NLM/NCBI)

Seufferheld and colleagues perform a phylogenetic analysis of the V-H^+ ^PPase domain, a component of the acidocalcisome in the eukaryotes and bacteria. They argue that the acidocalcisome was present in LUCA along with the V-H^+ ^PPase.

Critique:

The phylogenetic analysis of the V-H^+ ^PPases suggests a complicated evolutionary history with loss and possible lateral transfer playing a role in their distribution. However, reconstruction to LUCA is not strongly supported. Firstly this domain family is not widespread in the archaea. Further, the major archaeal branches (e.g. eury- and cren- archaea) also do not cluster with each other. Even within the bacteria, homologs from monophyletic groups do not group with each other. Given the presence of this family in diverse bacteria, I would find it more parsimonious to reconstruct the evolutionary history of this family as having evolved in the bacteria with subsequent lateral transfer to the archaea and eukaryotes.

The paragraph on the Pfam domains does not fit into the paper since these proteins show no domain complexity. Further, in this case the Pfam domain deposited in the database only captures the key catalytic motif of the protein. If the authors wish to discuss the domain in terms of sequence, they can write about the various transmembrane helices and the context of the catalytic residues.

Authors' Response: Our phylogenetic analyses of the V-H^+^PPase proteins include the amino acid sequence of the entire membrane-associated protein and not only of the domain regions. Furthermore, our phylogenetic tree describes the evolution of the sequence in 279 organisms spanning the three superkingdoms of life, Archaea (17 species), Bacteria (231), and Eukarya (31). These 279 sequences were selected from an analysis of a larger set of 776 sequences, following removal of paralogs or sequences with no genetic distance or that have no clear taxonomical descriptions. We find that the V-H^+^PPases are present in every one of the 776 organism analyzed and that the Pfam domain PF03030 is always present and captures the main sequence profile of the structure of these proteins. The claim of the reviewer that the domain is absent in Archaea and that its evolutionary history follows a complex series of losses and possible gains through LGT is therefore incorrect. The protein and associated domain is simply present everywhere. While variants could have been recruited by LGT and replaced, the protein was never lost in any lineage examined. We agree that LGT events are possible. They could have been responsible for the lack of phylogenetic signal that limits the correct grouping of organisms in the V-H^+ ^PPase tree. However, the topology of the tree has clearly defined groups that match taxonomical classification, including the Euryarchaeota, Proteobacteria, Thermotogales, Actinobacteria and Firmicutes. Some cases show non-monophyletic groupings that could have resulted from LGT. Nevertheless, it is important to notice that in several of these non-monophyletic groupings the phylogenetic support is very poor 0.5 to 0.76. While the tree of the vacuolar pyrophosphatase is one gene tree and has a largely star-like internal topology, it is significant that the phylogenetic reconstruction of this gene maintains essentially the three superkingdoms of life.

**Figure 3:**
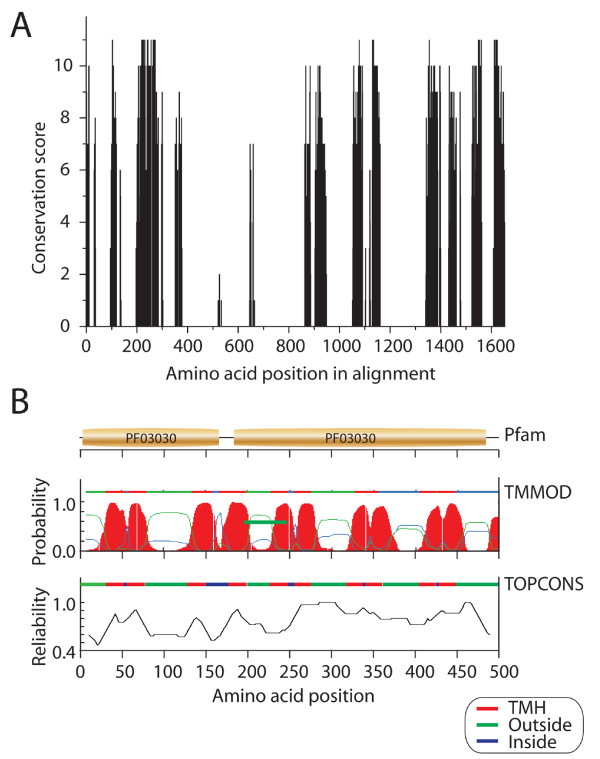
**Sequence conservation and transmembrane helical (TMH) make up of V-H**^**+**^**PPases**. (**A**) Sequence conservation of the set of aligned V-H^+^PPase sequences illustrated with sequence conservation scores obtained with JALVIEW. (**B**) Pfam domain definitions and TMH regions in the V-H^+^PPase of *R. rubrum*. The plots show TMH definitions obtained with TMMOD and TOPCONS. TMMOD TMH definitions are given together with posterior probabilities of the existence of a TMH region. The TOPCONS definitions are from a consensus

*An analysis of the mean distance between protein sequences using the JTT model with gaps excluded in the analysis showed that levels of sequence identity were 64.4%, much larger the 30% expected in the presence of high conservation levels. Using JALVIEW, we extracted conservation scores (in a scale from 0 to 11) for every nucleotide site with a proportion of gaps that is less than 25%. Analysis of the 523 sites (out of the total 1,652 in the alignment) that fulfills that criterion, shows the vast majority of sites have conservation scores above 7 (251 sites, 48%) and many have scores above 10 (90 sites, 17%) (Figure *3*). These results support the notion that V-H^+^PPases have highly conserved protein sequences and that the variable regions have little or highly degraded phylogenetic signal. This explains the star-like appearance and poorly supported nature of the V-H^+ ^PPase tree and suggests the V-H^+^PPase domain is old, and from a structural perspective, is highly canalized. This conclusion, together with its wide if not universal distribution in life, suggests that the most parsimonious explanation of the origin of this membrane-associated protein is in LUCA and not in any lineage of the diversified world of organisms, as the reviewer claims.*

*While a minority of sequences contain a duplicated PF03030 arrangement or are associated with another domain (PB008043), most contain a single Pfam domain. Thus, and as the reviewer correctly points out, our analyses show V-H^+^PPases do not exhibit a complex domain organization. Complex domain arrangements are the hallmark of recently evolved proteins in the protein world *[25]*. Consequently, the domain make up of V-H^+^PPases provides additional clues in support of their ancient origin. Remarkably, an analysis of trans-membrane helices (TMH) of the protein shows also high levels of structural conservation. The number of TMHs ranges 9-11with an average of 10.2 ± 0.75 (SD) and TMHs hold an average of 243.2 ± 20.30 amino acid residues. TMHs therefore represent on average 42.1 ± 3.46% of the protein. Figure *3*illustrates the position of the two PF03030 domain and the 10 TMHs in the sequence of R. rubrum YP426905, the model system for acidocalcisome studies. The V-H^+^PPases are therefore evolutionarily conserved at different levels, including sequence, domain structure, and TMH make-up, a fact that speaks about their ancient origins.*

### Reviewer's report 3

Daniel Kahn (Laboratoire de Biométrie et Biologie Évolutive, Université Lyon, France)

This reviewer provided no comments for publication

## Supplementary Material

Additional file 1 **Multiple alignments of PF03030 domain sequences distributed in the three superkingdoms (Archaea, Bacteria, and Eukarya)**. Shown above is the strongly conserved 57-residue region of the V-H^+^-PPase identified by Hedlund et al [53] and viewed using JALVIEW provided by the Pfam database. Click here for file

Additional file 2 **Taxa and accession numbers used in the phylogenetic analyses**. Click here for file
