# Supplementary material for: Evolution of vacuolar proton pyrophosphatase domains and volutin granules: clues into the early evolutionary origin of the acidocalcisome
Source: Biol Direct. 2011 Oct 5;6:50. doi: 10.1186/1745-6150-6-50 (PMC3198990; doi:10.1186/1745-6150-6-50)
Supplement: Additional file 1 — Multiple alignments of PF03030 domain sequences distributed in the three superkingdoms (Archaea, Bacteria, and Eukarya). Shown above is the strongly conserved 57-residue region of the V-H+-PPase identified by Hedlund et al [53] and viewed using JALVIEW provided by the Pfam database. [file 1745-6150-6-50-S1.PDF]

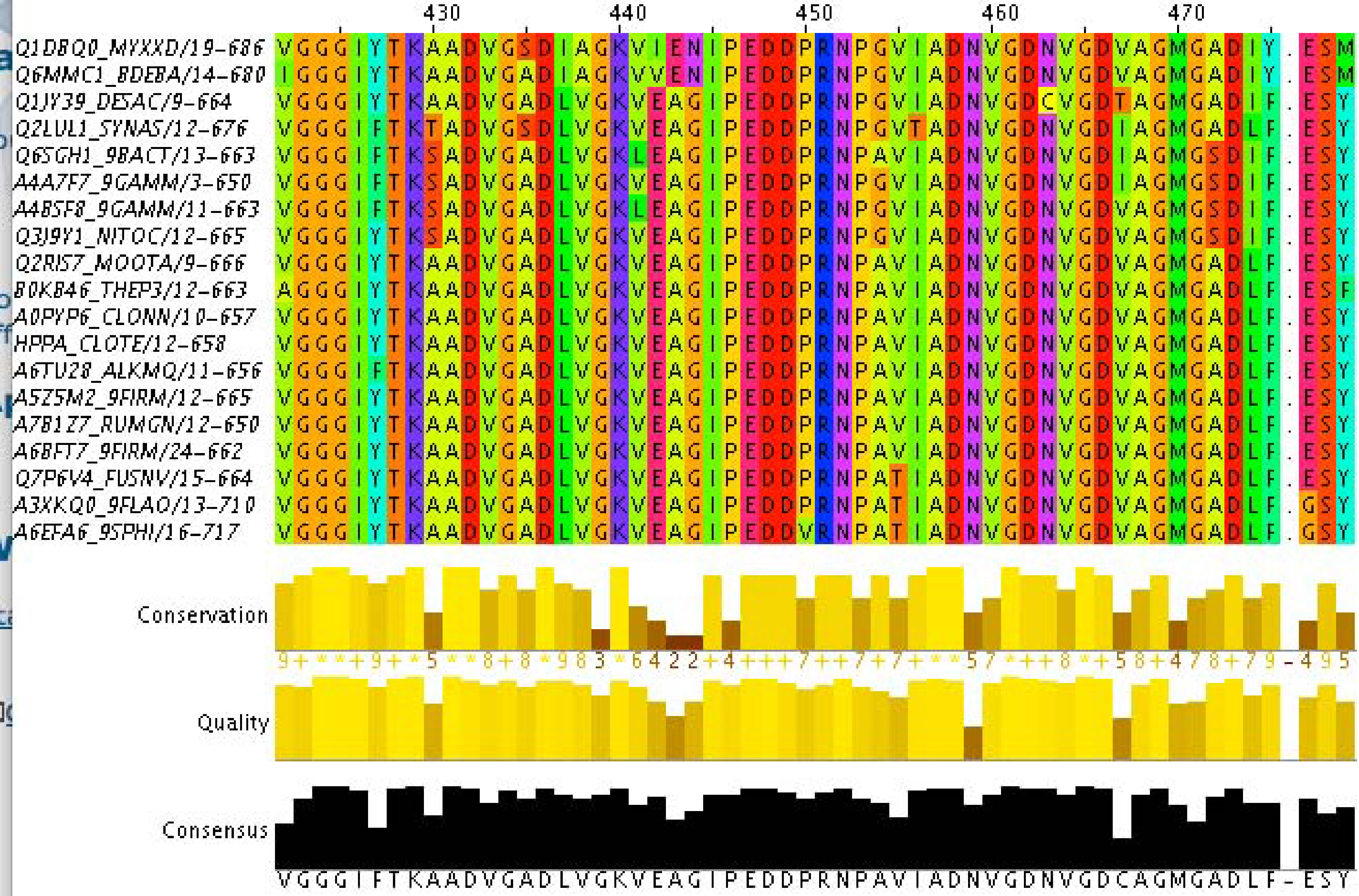
430
440
450
460
470
Q1DBQ0_MYXXD/19-686 VGGGIYTKAADVGSDIAGKVIENIPEDDPRNPGVIADNVGDNVGDVAGMGADIY.ESM
Q6MMC1_BDEBA/14-680 IGGGIYTKAADVGADIAGKVVENIPEDDPRNPGVIADNVGDNVGDVAGMGADIY.ESM
Q1JY39_DESAC/9-664 VGGGIYTKAADVGADLVGKVEAGIPEDDPRNPGVIADNVGDCVGDTAGMGADIF.ESY
Q2LUL1_SYNAS/12-676 VGGGIFTKTADVGSDLVGKVEAGIPEDDPRNPGVTADNVGDNVGDIAGMGADLF.ESY
Q6SGH1_9BACT/13-663 VGGGIFTKSADVGADLVGKLEAGIPEDDPRNPAVIADNVGDNVGDIAGMGSDIF.ESY
A4A7F7_9GAMM/3-650 VGGGIFTKSADVGADLVGKVEAGIPEDDPRNPGVIADNVGDNVGDIAGMGSDIF.ESY
A4BSF8_9GAMM/11-663 VGGGIFTKSADVGADLVGKLEAGIPEDDPRNPGVIADNVGDNVGDVAGMGSDIF.ESY
Q3J9Y1_NITOC/12-665 VGGGIYTKSADVGADLVGKVEAGIPEDDPRNPGVIADNVGDNVGDVAGMGSDIF.ESY
Q2RIS7_MOOTA/9-666 VGGGIYTKAADVGADLVGKVEAGIPEDDPRNPAVIADNVGDNVGDVAGMGADLF.ESY
B0KB46_THEP3/12-663 AGGGIYTKAADVGADLVGKVEAGIPEDDPRNPAVIADNVGDNVGDVAGMGADLF.ESF
A0PYP6_CLONN/10-657 VGGGIYTKAADVGADLVGKVEAGIPEDDPRNPAVIADNVGDNVGDVAGMGADLF.ESY
HPPA_CLOTE/12-658 VGGGIYTKAADVGADLVGKVEAGIPEDDPRNPAVIADNVGDNVGDVAGMGADLF.ESY
A6TU28_ALKMQ/11-656 VGGGIFTKAADVGADLVGKVEAGIPEDDPRNPAVIADNVGDNVGDVAGMGADLF.ESY
A5Z5M2_9FIRM/12-665 VGGGIYTKAADVGADLVGKVEAGIPEDDPRNPAVIADNVGDNVGDVAGMGADLF.ESY
A7B1Z7_RUMGN/12-650 VGGGIYTKAADVGADLVGKVEAGIPEDDPRNPAVIADNVGDNVGDVAGMGADLF.ESY
A6BFT7_9FIRM/24-662 VGGGIYTKAADVGADLVGKVEAGIPEDDPRNPAVIADNVGDNVGDVAGMGADLF.ESY
Q7P6V4_FUSNV/15-664 VGGGIYTKAADVGADLVGKVEAGIPEDDPRNPATIADNVGDNVGDVAGMGADLF.ESY
A3XKQ0_9FLAO/13-710 VGGGIYTKAADVGADLVGKVEAGIPEDDPRNPATIADNVGDNVGDVAGMGADLF.GSY
A6EFA6_9SPHI/16-717 VGGGIYTKAADVGADLVGKVEAGIPEDDVRNPATIADNVGDNVGDVAGMGADLF.GSY
Conservation
9+**+9+*5**8+8*983*6422+4+++7++7+7+**57*++8*+58+478+79-495
Quality
Consensus
VGGGIFTKAADVGADLVGKVEAGIPEDDPRNPAVIADNVGDNVGDCAGMGADLF-ESY
