# Supplementary material for: Evolution of vacuolar proton pyrophosphatase domains and volutin granules: clues into the early evolutionary origin of the acidocalcisome
Source: Biol Direct. 2011 Oct 5;6:50. doi: 10.1186/1745-6150-6-50 (PMC3198990; doi:10.1186/1745-6150-6-50)
Supplement: Additional file 2 — Taxa and accession numbers used in the phylogenetic analyses. [file 1745-6150-6-50-S2.PDF]

Additional File 2. Taxa and accession numbers used in the phylogenetic analyses

TAXA	Accession numbers
Rhizobium etli	Q2KA06
Rhizobium leguminosarum	Q1MIT1
Agrobacterium radiobacter	B9JCY4
Agrobacterium tumefaciens	Q8UG67
Rhizobium meliloti	Q8VRZ3
Sinorhizobium medicae	A6U7Q0
Agrobacterium vitis	B9JV82
Brucella abortus	B2S519
Brucella canis	A9MAF1
Brucella ceti	C0G5N5
*Brucella melitens*is	Q8YGH4
Brucella suis	B0CL96
Brucella ovis	A5VPV3
Ochrobactrum intermedium	C4WFQ4
Ochrobactrum anthropi	Q8VRZ2
Rhizobium loti	Q983A3
Methylocella silvestris	B8ENB6
Beijerinckia indica	B2ICH6
Aurantimonas manganoxydans	ZP_01227325
Stappia aggregata	ZP_01545402
Labrenzia aggregata	A0NMF0
Labrenzia alexandrii	B9QV43
Bradyrhizobium japonicum	Q89K83
Oligotropha carboxidovorans	B6JGI6
Nitrobacter hamburgensis	Q1QLC2
Nitrobacter winogradskyi	Q3SRT3
Rhodopseudomonas palustris	YP_532535
Xanthobacter autotrophicus	YP_001419075
Azorhizobium caulinodans	A8IBH1
Methylobacterium chloromethanicum	B7KUJ9
Methylobacterium extorquens	C5AXH1
Methylobacterium populi	B1ZIM5
Methylobacterium radiotolerans	B1LZA8
Methylobacterium nodulans	B8IPI6
Caulobacter crescentus	NP_420176
Phenylobacterium zucineum	B4RB24
Maricaulis maris	YP_756758

Oceanicaulis alexandrii	A3UCC6
Hyphomonas neptunium	Q0C316
Parvibaculum lavamentivorans	A7HX90
Pelagibacter ubique	Q4FLU2
Candidatus Pelagibacter	Q1UZ93
Rhodospirillum rubrum	YP_426905
Rhodospirillum centenum	B6IMS1
Novosphingobium aromaticivorans	Q2G9G1
Erythrobacter litoralis	YP_457403
Sphingopyxis alaskensis	YP_617067
Sphingomonas wittichii	YP_001263265
Magnetospirillum magneticum	Q2W4S3
Magnetospirillum gryphiswaldense	A4TZI8
Lawsonia intracellularis	YP_594877
Teredinibacter turnerae	C5BMT0
Verminephrobacter eiseniae	A1WM79
Acidovorax avenae	YP_004235012
Acidovorax ebreus	B9MA49
Polaromonas naphthalenivorans	A1VMY6
Variovorax paradoxus	C5CYM5
Leptothrix cholodnii	B1XZ65
Methylibium petroleiphilum	A2SHN8
Rhodoferax ferrireducens	Q21YE8
Herminiimonas arsenicoxydans	A4G7X7
Oxalobacter formigenes	C3X6L8
Xanthomonas campestris	NP_638658
Xanthomonas axonopodis	Q8PH20
Stenotrophomonas maltophilia	B2FT52
Vesicomyosocius okutanii	A5CXT3
Ruthia magnifica	A1AVD4
Gemmatimonas aurantiaca	C1A776
Thiobacillus denitrificans	Q3SIS2
Nitrosospira multiformis	Q2YB25
Nitrosomonas eutropha	YP_747021
Geobacter lovleyi	B3E4Y7
Geobacter sulfurreducens	Q747H5
Geobacter metallireducens	Q39QM2
Geobacter bemidjiensis	YP_002137390
Geobacter uraniireducens	A5GC38
Pelobacter propionicus	A1ATG4
Desulfococcus oleovorans	A8ZXZ6
Desulfatibacillum alkenivorans	B8FLB6
Chthoniobacter flavus	B4CVZ9
Methylacidiphilum infernorum	B3DZS9
Thermomicrobium roseum	B9KYZ6
Methylococcus capsulatus	Q609P4

Syntrophobacter fumaroxidans	A0LML7
Desulforudis audaxviator	B1I0V1
Methanococcoides burtonii	YP_565684
Kordia algicida	A9DJK7
Gramella forsetii	A0LY38
Dokdonia donghaensis	A2TSB0
Croceibacter atlanticus	YP_003715950
Polaribacter irgensii	A4BZY9
Flavobacterium johnsoniae	A5FJW6
Leeuwenhoekiella blandensis	ZP_01060378
Amoebophilus asiaticus	B3ES25
Chryseobacterium gleum	C0YJW4
Microscilla marina	A1ZEZ2
Acetabularia mediterranea	BAA83103
Acetabularia acetabulum	Q9STC8
Robiginitalea biformata	YP_003196855
Prosthecochloris vibrioformis	A4SE64
Elodictyon luteolum	Q3B3L7
Chlorobaculum parvum	B3QP07
Prosthecochloris aestuarii	B4S8L2
Chloroherpeton thalassium	B3QVV1
Plesiocystis pacifica	A6GBL9
Hordeum brevisubulatum	Q84QI7
Triticum aestivum	A9LRZ1
Hordeum vulgare	Q9FS12
Chenopodium rubrum	Q8L5B2
Chenopodium glaucum	Q1W2P4
Halostachys caspica	A4LAP4
Kalidium foliatum	A1E9B0
Phaseolus aureus	P21616
Vigna radiata	O22124
Medicago truncatula	B6DXD7
Pyrus communis	Q8GT22
Cucurbita moschata	BAA33149
Hevea brasiliensis	Q6R4U3
Nicotiana rustica	Q197Z6
Thellungiella salsuginea	Q6T553
Malus domestica	A9X9A3
Zygophyllum xanthoxylum	A7XY78
Picea sitchensis	C0PRN4
Chara corallina	Q9ZWI8
Chlamydomonas reinhardtii	CAC44451
Leishmania infantum	A4I6P8
Leishmania major	Q4Q6E1
Leishmania braziliensis	A4HJA5
Trypanosoma cruzi	Q9NDF0

Tetrahymena thermophila	XP_001011583
Toxoplasma gondii	AAK38077
Leptospira borgpetersenii	Q04U06
Leptospira interrogans	YP_002219
Leptospira biflexa	B0S8X5
Salinibacter ruber	Q2S4D3
Aciduliprofundum boonei	B5IDA5
Bacteroides ovatus	A7LVP2
Bacteroides caccae	A5ZKN0
Bacteroides fragilis	Q5LIL5
Bacteroides xylanisolvens	CBK67775
Bacteroides thetaiotaomicron	Q8A294
Bacteroides intestinalis	B3C7L3
Bacteroides uniformis	A7UYB2
Bacteroides stercoris	B0NSQ0
Bacteroides eggerthii	B7ALD5
Bacteroides dorei	C3R6Q7
Bacteroides vulgatus	A6L2M4
Bacteroides plebeius	B5CYN8
Bacteroides coprocola	B3JI04
Parabacteroides merdae	A7AD83
Parabacteroides distasonis	A6LIE5
Akkermansia muciniphila	B2ULG2
Verrucomicrobiae bacterium	B5JQT8
Blastopirellula marina	A3ZRC4
Planctomyces maris	A6BZZ1
Candidatus Kuenenia	Q1PZR6
Candidatus Cloacamonas	B0VHS9
Brachyspira hyodysenteriae	C0R142
Clostridium phytofermentans	A9KSE9
Clostridium leptum	A7VNH8
Clostridium methylpentosum	C0EI34
Bacteroides capillosus	A6NPF7
Shuttleworthia satelles	C4GBA7
Thermotoga petrophila	A5IKP7
Marinitoga piezophila	B7RDQ4
Thermotoga naphthophila	YP_003346312
Thermotoga maritima	Q9S5X0
Thermotoga neapolitana	Q5CBQ3
Thermosipho melanesiensis	A6LP21
Thermosipho africanus	B7IEX6
Thermotoga lettingae	A8F6U1
Petrotoga mobilis	YP_001568076
Bacteroides pectinophilus	B7ASN2
Eubacterium eligens	C4Z6Y1
Kosmotoga olearia	C5CIC6

Dictyoglomus turgidum	B8E0W5
Dictyoglomus thermophilum	B5YF34
Fervidobacterium nodosum	A7HMQ6
Coprothermobacter proteolyticus	B5Y802
Pyrobaculum aerophilum	NP_559532
Pyrobaculum arsenaticum	A4WIY1
Pyrobaculum islandicum	A1RV93
Pyrobaculum calidifontis	A3MTK8
Thermoproteus tenax	A9JQI4
Caldivirga maquilingensis	YP_001540035
Nitrosopumilus maritimus	A9A1E8
Cenarchaeum symbiosum	A0RW83
Clostridium thermocellum	YP_001037849
Nitrosococcus oceani	Q3J9Y1
Nitrococcus mobilis	A4BSF8
Methylophaga thiooxidans	C0N833
Congregibacter litoralis	A4A7F7
Pelobacter carbinolicus	Q3A315
Desulfuromonas acetoxidans	Q1JY39
Myxococcus xanthus	YP_629923
Bdellovibrio bacteriovorus	Q6MMC1
Anaerococcus lactolyticus	C2BHH2
Anaerococcus tetradius	C2CHX6
Anaerococcus hydrogenalis	B6W7C5
Abiotrophia defectiva	C4G773
Clostridium novyi	A0PYP6
Clostridium tetani	Q898Q9
Clostridium butyricum	C4IDY6
Alkaliphilus oremlandii	YP_001512518
Alkaliphilus metalliredigens	YP_001321355
Ruminococcus lactaris	B5CSC0
Dorea formicigenerans	B0GAF9
Coprococcus comes	C0B9C7
Ruminococcus gnavus	A7B1Z7
Clostridium hylemonae	C0C4K6
Clostridium scindens	B0NDR2
Dorea longicatena	A6BFT7
Eubacterium hallii	C0EVH8
Blautia hydrogenotrophica	C0CPB6
Oribacterium sinus	C2KZ86
Pelotomaculum thermopropionicum	YP_001213266
Desulfotomaculum reducens	YP_001114313
Carboxydothermus hydrogenoformans	YP_359158
Heliobacterium modesticaldum	B0TGJ8
Desulfitobacterium hafniense	YP_521069
Natranaerobius thermophilus	B2A6Y9

Dethiobacter alkaliphilus	C0GFB8
Halothermothrix orenii	B8CYF6
Methanoculleus marisnigri	YP_001045961
Methanosarcina acetivorans	NP_618750
Methanocorpusculum labreanum	YP_001029450
Methanospirillum hungatei	YP_503835
Thermoanaerobacter mathranii	YP_003676510
Thermoanaerobacter pseudethanolicus	B0KB46
Anaerofustis stercorihominis	B1C820
Finegoldia magna	B0S160
Parvimonas micra	A8SMK6
Ostreococcus lucimarinus	A4RRD6
Symbiobacterium thermophilum	Q67L99
Eubacterium ventriosum	A5Z5M2
Anaerostipes caccae	B0M926
Fusobacterium nucleatum	ZP_04969641
Caldicellulosiruptor saccharolyticus	A4XHY4
Caldicellulosiruptor kronotskyensis	YP_004024560
Anaerocellum thermophilum	B9MQ79
Carboxydibrachium pacificum	B7R706
Thermoanaerobacter tengcongensis	Q8RCX1
Clostridium beijerinckii	A6M3H6
Methanosphaerula palustris	B8GJH9
Methanoregula boonei	A7I486
Acidobacterium capsulatum	C1F569
Plasmodium chabaudi	Q4Y125
Cyanidioschyzon merolae	Q4AC87
Korarchaeum cryptofilum	B1L5G5
Chloroflexus aurantiacus	Q8VNW3
Chloroflexus aggregans	YP_002463789
Roseiflexus castenholzii	A7NPW2
Elusimicrobium minutum	B2KCY8
Syntrophomonas wolfei	Q0AY30
Clostridium cellulolyticum	B8I4X7
Azobacteroides pseudotrichonymphae	B6YQJ7
Opitutus terrae	B1ZTP5
Candidatus Koribacter versatilis	YP_590993
Solibacter usitatus	Q023J7
Chlorobium ferrooxidans	Q0YT89
Pelodictyon phaeoclathratiforme	B4SGM2
Chlorobium tepidum	Q8KDT8
Stigmatella aurantiaca	Q08ZE0
Mobiluncus mulieris	C2KPK8
Propionibacterium acnes	Q6A7G5
Beutenbergia cavernae	C5BY02
Kineococcus radiotolerans	YP_001360239

Saccharopolyspora erythraea	YP_001102649
Salinispora arenicola	A8M5X3
Salinispora tropica	A4XC03
Acidothermus cellulolyticus	A0LWD5
Streptomyces coelicolor	Q6BCL0
Streptomyces sviceus	ZP_06918746
Streptomyces avermitilis	NP_825793
Streptomyces pristinaespiralis	B5HA91
Streptomyces clavuligerus	B5GPP4
Streptomyces griseus	B1VM24
Thermobifida fusca	YP_288195
Frankia alni	YP_716705
